# Applications of near-infrared spectroscopy in “anaerobic” diagnostics – SmO_2_ kinetics reflect PCr dephosphorylation and correlate with maximal lactate accumulation and maximal pedalling rate

**DOI:** 10.5114/biolsport.2023.122481

**Published:** 2023-03-03

**Authors:** Anna Katharina Dunst, Christian Manunzio, Andri Feldmann, Clemens Hesse

**Affiliations:** 1 Institute for Applied Training Science, Leipzig, Germany; 2 University Hospital Bonn, Children’s Hospital, Department of Paediatric Cardiology, Sports Clinic, Bonn, Germany; 3 University of Bern, Institute of Sport Science, Bern, Switzerland; 4 German Cycling Federation, Frankfurt/Main, Germany

**Keywords:** Track cycling, Performance modeling, NIRS, Maximal sprint, Exercise physiology, MOXY

## Abstract

We investigated the relationship of the time-dependent behaviour of muscle oxygen saturation SmO_2_(t), phosphagen energy supply W_PCr_(t) and blood lactate accumulation ΔBLC(t) during a 60-s all-out cycling sprint and tested SmO_2_(t) for correlations with the end of the fatigue-free state t_Ff_, maximal pedalling rate PR_max_ and maximal blood lactate accumulation rate v̇La_max_. Nine male elite track cyclists performed four maximal sprints (3, 8, 12, 60 s) on a cycle ergometer. Crank force and cadence were monitored continuously to determine PR_max_ and t_Ff_ based on force-velocity profiles. SmO_2_ of the vastus lateralis muscle and respiratory gases were measured until the 30^th^ minute after exercise. W_PCr_ was calculated based on the fast component of the post-exercise oxygen uptake for each sprint. Before and for 30 minutes after each sprint, capillary blood samples were taken to determine the associated ΔBLC. Temporal changes of SmO_2_, W_PCr_ and ΔBLC were analysed via non-linear regression analysis. v̇La_max_ was calculated based on ΔBLC(t) as the highest blood lactate accumulation rate. All models showed excellent quality (R^2^ > 0.95). The time constant of SmO_2_(t) τ_SmO2_ = 2.93 ± 0.65 s was correlated with the time constant of W_PCr_(t) τ_PCr_ = 3.23 ± 0.67 s (r = 0.790, p < 0.012), v̇La_max_ = 0.95 ± 0.18 mmol · l^−1^ · s^−1^ (r = 0.768, p < 0.017) and PR_max_ = 299.51 ± 14.70 rpm (r = -0.670, p < 0.049). t_Ff_ was correlated with τ_SmO2_ (r = 0.885, p < 0.001). Our results show a time-dependent reflection of SmO_2_ kinetics and phosphagen energy contribution during a 60-s maximal cycling sprint. A high v̇La_max_ results in a reduction, a high PR_max_ in an increase of the desaturation rate. The half-life of SmO_2_ desaturation indicates the end of the fatigue-free state.

## INTRODUCTION

As an approach to the assessment of training and performance, the kinetics of skeletal muscle oxygenation are being examined more and more extensively in the laboratory and the field [1]. The application of the Beer-Lambert law to near-infrared spectroscopy (NIRS) allows for non-invasive and convenient measurement of changes in local muscle tissue oxygenation [2–4]. The procedure has been validated for use during dynamic exercise in adults [5], so that the NIRS signal obtained during exercise can be used to estimate the relationship between local O_2_ supply and demand (SmO_2_) or to describe changes in tissue blood volume at the site of O_2_ exchange via changes in total haemoglobin and myoglobin mass (tHb) [6].

Since the recovery of phosphocreatine (PCr) levels in muscle following activity or ischaemia depends primarily on production of ATP by mitochondria, which, in turn, depends on oxygen availability [7–10], oxygen supply and demand as reflected in NIRS and the levels of high-energy phosphates are closely correlated [3, 4]. For example, in muscles at rest, a relationship between dephosphorylation of PCr and desaturation has been demonstrated. Utilizing NIRS and magnetic resonance spectroscopy (MRS) in combination, Hamaoka and colleagues [11] observed that arterial occlusion resulted in an exponential decrease in SmO_2_. Upon reaching desaturation, the level of PCr decreased linearly, a phenomenon proposed to be required for maintenance of ATP levels.

The observed delay between SmO_2_ desaturation and the reduction in PCr levels in muscle under ischaemic conditions at rest results from the interaction of different systems of energy supply. At rest, energy requirements are usually met almost exclusively by mitochondrial respiration. Subsequent transport of this energy from the mitochondria to the sarcomere involves a potential gradient created by rephosphorylation of PCr [10, 12–14]. Under ischaemic conditions at rest, the level of PCr in muscle remains stable until decreasing availability of oxygen lowers the rate of mitochondrial energy production; thereafter, the rate of PCr dephosphorylation reflects the energy consumption.

Boushel et al. [15] reported a two-fold higher rate of SmO_2_ desaturation during exercise than at rest under ischaemic conditions and attributed this to a greater demand for energy in combination with glycolysis. However, the exact relationship between SmO_2_, the level of PCr and glycolysis during maximal dynamic exercise, e.g., maximal bouts of cycling, remains to be elucidated. Under such conditions, we would predict a close temporal correlation between the kinetics of SmO_2_ desaturation and PCr dephosphorylation, which could be influenced by the rate of glycolysis.

During exercise, the rate of ATP resynthesis is determined by the phosphorylation of ADP by enyzmatic dephosphorylation of PCr in combination with glycolytic and mitochondrial ATP production [12, 16]. For instance, during twitch contractions, loss of ATP is buffered through usage of PCr by creatine kinase to phosphorylate ADP [13, 17]. Following such a contraction, the level of PCr recovers more rapidly than during the final period of stimulation, indicating the involvement of different mechanisms [17]. Chung and co-workers [17] reported that during exercise the phosphorylation status is closely coupled to the rate of glycolysis and oxidative phosphorylation. Assuming unchanged levels of creatine kinase activity, rephosphorylation of PCr depends on these two systems and deficient rephosphorylation leads to compensatory increases in mitochondrial and glycolytic ATP production [12, 17]. When the rate of ATP consumption changes suddenly, glycolysis, which occurs in the sarcoplasm, contributes more rapidly to the ADP/ATP gradient than mitochondrial respiration does [9]. During the initial phase of muscle contraction, an increase in oxidative phosphorylation is limited until the cardiopulmonary system has adapted to supply the greater amount of oxygen in demand [18, 19].

Consequently, during the first seconds of maximal exercise, the aerobic contribution to energy production is likely to be relatively small, and PCr is restored predominantly by glycolysis. As glycolysis is sensitive to pH and ATP hydrolysis during maximal exercise leads to a rapid reduction in muscle pH [20], the glycolytic flux may decline within a few seconds [12], whereas mitochondrial ATP synthesis increases with time [19]. In addition, acidic conditions also favour dissociation of O_2_ [19].

When sprinting maximally, the PCr consumed is rephosphorylated by creatine kinase utilizing ATP produced by glycolysis and mitochondrial respiration. From the increase in the rate of glycolysis and associated production of lactate within seconds after the start of exercise [21, 22] it could be concluded that oxidation of lactate is likely to play a key role in energy equilibrium and, thereby, in SmO_2_ kinetics. We hypothesize that during maximal sprint exercise, the kinetics of SmO_2_ desaturation reflect those of PCr dephosphorylation and are correlated with the maximal rate of lactate accumulation v̇La_max_ as an indirect parameter of maximal glycolytic activity.

Maximal energy flow can only be generated until PCr stores are reduced to a maximum of half [23]. The time span for maximal energy flow and maximal power output seems to be well reflected by the half-life of PCr dephosphorylation, which is a function of its time constant. In our recent work, we presented a new approach to determine the end of the time interval of maximal energy flow (fatigue-free time span) the timespan up to the first systematic deviation from the fatigue-free force-velocity (F/v) profile (t_Ff_) [24]. Assuming a temporal overlap of the SmO_2_ desaturation and PCr dephosphorylation kinetics, a correlation between t_Ff_ and the time constant of SmO_2_ can be hypothesised.

Further assuming a correlation between the maximal rate of energy, the rate of PCr dephosphorylation and muscle fibre type composition [25], with maximal pedalling rate being dependent on the proportion of fast-twitch muscle fibres in the muscles involved [26], we also predict an increase in the rate of PCr dephosphorylation and of SmO_2_ desaturation with increasing pedalling rate. According to the model presented, direct measurement of SmO_2_ should provide a powerful approach to assessment of changes in the levels of high-energy phosphates and lactate with time.

## MATERIALS AND METHODS

### Participants

Nine male (21.1 ± 3.4 years, 184.9 ± 5.1 cm, 89.6 ± 6.4 kg, BFP: 13.4 ± 1.7% , V̇O_2max_: 53.9 ± 4.6 ml kg^−1^ min^−1^, P_max_: 1744 ± 111 W) (mean ± standard deviation) elite track cycling sprinters took part in this study. Since our study design required high-level neuromuscular and metabolic performance, only athletes who had demonstrated closely linear F/v profiles (R^2^ > 0.95) in previous tests and who had shown constant performance across all races per day in track cycling sprint events at international championships were included.

Participants were requested to refrain from consuming alcohol and from intense training and asked to maintain their normal drinking and eating habits 24 h prior to the experimental session. All participants provided written informed consent for participation in this study. The study was approved by the institute’s ethical committee and performed in accordance with the Declaration of Helsinki.

### Exercise protocol

Each participant visited the laboratory once, completing four maximal sprints in a seated position on a cycle ergometer. As the testing protocol was similar to the daily training and competition among these track cyclists, who had also previously carried out similar performance tests in the laboratory, no additional test familiarisation was considered necessary. All participants used their own cycling shoes and pedals during these efforts. The ergometer settings resembled actual competition demands.

The participants warmed up before each sprint with 6 minutes of low-intensity cycling (1–1.5 W · kg^−1^ body weight), followed by a 3-s maximal sprint. Participants rested passively for 10 minutes between warm-up and testing. Four maximal sprints (3, 8, 12, 60 s) were performed in an isokinetic mode at a cadence of 120 rpm on an SRM cycle ergometer (Schoberer Rad Messtechnik GmbH, Jülich, Germany) with a 9-kg flywheel, in 7^th^ gear. With these settings, the pedal force decreased linearly during the initial acceleration phase and the target cadence of 120 rpm was reached after 3–4 s.

The participants were asked to accelerate as rapidly as possible from a simulated rolling start at approximately 20 rpm to 120 rpm at a self-selected time-point and to continue sprinting with maximal effort until the end of each sprint, with the investigators providing emphatic verbal encouragement throughout the entire test. 30 min after the 12-s sprint, each participant performed 6 s of maximal-cadence, low-resistance cycling (the motoric test) on an ergometer [24]. The settings enabled the athletes to reach a cadence of ≥ 160 rpm within the first 3 s, which generated additional data in the high-frequency cadence range. Combination of these data with the fatigue-free rate of pedalling during the acceleration phase of each sprint allowed more valid determination of the F/v profiles [27].

Tests were separated by approximately 2 hours, during which the athletes were asked to eat a light meal, corresponding to the individual’s calculated energy expenditure during the preceding test. The next test began when comparable levels of blood lactate and blood glucose were observed.

### Equipment and Measurements

Data collection started from 3 minutes before the beginning of the standardized warm-up and ended 30 minutes after exercise, while participants remained seated, with as little movement as possible.

A commercially available continuous-wave NIRS device was used (Moxy Monitor; Fortiori Designs LLC, MN, USA) to continuously measure SmO_2_ and tHb (total mass of haemoglobin and myoglobin) of the vastus lateralis muscle (VL), as this muscle has been used most commonly in recent studies using NIRS in cyclists [1]. The sensors were fixed in place using medical adhesive tape (Hypafix; BSN Medical, DE) and covered with a compatible commercially available light shield to eliminate possible ambient light intrusion. The device uses four wavelengths (680, 720, 760 and 800 nm) to assess absorbency via the modified Beer-Lambert law resulting in a relative concentration of SmO_2_ as a percentage in the following equation: oxygenated Hb + Mb / oxygenated Hb + Mb + deoxygenated Hb + Mb = SmO_2_ [28]. The device detectors are spaced at 12.5 mm and 25 mm from the emitter. The sampling rate was set to 0.5 Hz, which samples the four wavelengths over 10 cycles for an averaged output every second and gathered using the Idiag Moxy software (Idiag AG, CH). With the emitter-detector spacing of 25 mm, a penetration depth of 15.0 mm can be expected (ibid.). The adipose tissue thickness (ATT) of the subjects at the measurement site was individually measured and none breached the upper threshold of 15.0 mm; therefore ATT interference is considered negligible.

Net crank torque (M) and angular velocity (ω) were monitored continuously at a sample rate of 500 Hz with an SRM power meter (Schoberer Rad Messtechnik GmbH, Jülich, Germany). The average data from units of 10 Hz were used for calculations.

Before and directly after each sprint, as well as 1, 3, 5, 7, 10, 15, 20, 25 and 30 minutes after the 8- and 12 s-sprints, 20 µl of capillary blood was collected from the hyperaemic ear lobe for haemolysis and enzymatic-amperometric determination of lactate and glucose (Biosen, EKF Diagnostics, Magdeburg, Germany). Since accumulation of blood lactate during the 3-s test was expected to be limited and the participants had already performed a considerable number of such samplings, capillary blood was only collected during the first, third, fifth and tenth minutes following this sprint to ensure sufficient blood flow, prevent sample contamination and enhance comfort.

Respiratory gases were measured continuously with a breath-by-breath portable gas analyser (Metamax 3B, Cortex, Leipzig/Germany). Prior to each test the gas analyser was calibrated using a known concentration of gases and a 3-litre syringe following the manufacturer’s recommendations.

### Data processing

Raw data from the ergometer were obtained at a sample rate of 10 Hz. From these values, the mean tangential force F at both pedals, averaged over one revolution (taking into account individual crank length), as well as the corresponding pedalling rate, was derived.

To determine fatigue-free maximal pedalling rate, the fatigue-free force-velocity (F/v) profile was created based on the pedalling rate and corresponding mean crank force during the first 3–4 cycles of pedalling (≤ 3 s in duration, data point in the cadence range from 30 to 120 rpm) in each sprint, during which the crank force decreased linearly. As recommended by Dunst et al. [27, 29] 1 or 2 revolutions with very high pedalling rate (data point in the cadence range ≥ 160 rpm) attained during the first 3 s of the motoric test and its corresponding mean crank force were incorporated into these calculations. The data points with the best F/v relationship were selected. Since pedalling rate is directly proportional to the tangential speed of motion at the pedal, the F/v profile was based on pedalling rate (as specific movement velocity) and corresponding mean crank force F [29]. Parameters of the linear model function

F(v)=a⋅v+b (1),

were determined by linear regression analysis and characterise the fatigue-free relationship between mean pedal force (N) and specific movement velocity v (rpm) (ibid.). Maximal pedalling rate was calculated as PR_max_ = -b a^−1^.

With the onset of fatigue, reflected in reduced energy production by the main propulsion muscles, the mean pedal force decreases at each cadence so that the F/v profile at time t F(v,t) is lower than the fatigue-free maximum F(v). We defined the timespan up to the first systematic deviation from F(v) by the time-point t_Ff_ after which F(v,t) fell below and thereafter never returned to the fatigue-free maximal F(v) [24]:

$${\text{t}}_{\text{Ff}}\text{:=min}\{\tilde{\text{t}}\in \left[\text{s};\text{T}\right]|\text{F}\left(\text{v},\text{t}\right)\text{<F}\left(\text{v}\right)\forall \text{t}\in \left[\text{s};\text{T}\right]:\text{t>}\tilde{\text{t}}\},$$ (2),

where s=0 represents the initiation and T the termination of each performance test

Parameters of the exponential model function of SmO_2_(t) were determined by non-linear regression analysis based on the SmO_2_ data of the participants’ 60-s-sprint [30]:

$$\text{Sm}{\text{O}}_{2}\left(\text{t}\right)=\text{Sm}{\text{O}}_{2\text{Base}}-\text{Sm}{\text{O}}_{2\text{A}}\cdot (1-{\text{e}}^{\frac{-\text{t}+\text{TD}}{{}^{\text{τ}}\text{Sm}{\text{O}}_{2}}})$$ (3),

with SmO_2base_ as the resting level before the start of a sprint, SmO_2A_ as the amplitude of the Sm_O2_ drop and τ_SmO2_ as the time constant of desaturation.

For each sprint test, excess post-exercise oxygen consumption (V̇O_2EPOC_ ) was determined by non-linear regression analysis using the following bi-exponential 4-parameter model to establish the phosphagen energy supply [31]:

$$\dot{\text{V}}{\text{O}}_{2\text{EPOC}}\left(\text{t}\right)={\text{A}}_{\text{FC}}\cdot {\text{e}}^{\frac{-\text{t}+\text{TD}}{{}^{\text{τ}}\text{a}}}+{\text{B}}_{\text{SC}}\cdot {\text{e}}^{\frac{-\text{t}+\text{TD}}{{}^{\text{τ}}\text{b}}}+\dot{\text{V}}{\text{O}}_{2\text{Base}}$$ (4),

where A_FC_ and B_SC_ represent the amplitudes of the fast component (associated with post-exercise resynthesis of creatine phosphate) and the slow component (associated with post-exercise lactate oxidation), τa and τb the corresponding time constants and V̇O_2Base_ the asymptotic resting oxygen uptake at time →∞.

For equations (3) and (4), a time delay (TD) was used to compensate for any delays in the measurement data.

Replenishment of high-energy phosphates was estimated based on the product of the amplitude A_FC_ and time constant τ_a_ of the fast component (V̇O_2PCr_ = A_FC_τ_a_). Phosphagen energy supply was calculated from the latter by [16, 32]:

$${\text{W}}_{\text{PCr}}={\text{VO}}_{\text{2PCr}}\cdot \text{CE}$$ (5),

employing a caloric equivalent (CE) of 20.9 J ml^-1^ [16].

Based on W_PCr_ of the single sprint tests, time-dependent energy contribution of the phosphagen energy system during the 60-s test was analysed with the following mono-exponential 2-parameter model by non-linear regression analysis:

$${\text{W}}_{\text{PCr}}\left(\text{t}\right)={\text{W}}_{\text{PCrTOT}}\cdot (1-{\text{e}}^{\frac{-\text{t}}{{}^{\text{τ}}\text{PCr}}})$$ (6),

where W_PCrTOT_ represents the maximum of phosphatic energy supply at time →∞ and τ_PCr_ represents the corresponding time constant.

Since the time-point when maximal blood lactate concentration occurs is unpredictable, the dynamics of blood lactate response were calculated by non-linear regression analysis using a 3-parameter-model to determine maximal blood lactate concentration [33, 34]:

$$\text{BLC}\left(\text{t}\right)=\frac{\text{A}\cdot {\text{k}}_{1}}{{\text{k}}_{1}-{\text{k}}_{2}}\cdot \left({\text{e}}^{{\text{-k}}_{\text{1}}\cdot \text{t}}{\text{-e}}^{{\text{-k}}_{2}\cdot \text{t}}\right)+\text{BLC}\left(0\right)$$ (7),

with extra-vascular increase (A) and rate constants of BLC appearance (k_1_) and disappearance (k_2_). For each sprint test, corresponding ΔBLC (mmol · l^−1^) was calculated by subtraction of the maximum of BLC(t) and pre-exercise blood lactate (BLC_max_-BLC(0)) as described by Mader [35].

Following the model function of lactate formation rate reported by Mader [35, 36], the time-dependent ΔBLC was analysed by non-linear regression analysis using the following 3-parameter bi-exponential model:

$$\Delta \text{BLC}\left(\text{t}\right)=\text{B}\cdot (\text{-}\frac{\text{1}}{{\text{k}}_{3}}{\text{e}}^{{\text{-k}}_{3}\cdot \text{t}}+\frac{\text{1}}{{\text{k}}_{4}}{\text{e}}^{{\text{-k}}_{4}\cdot \text{t}})+\text{C}$$ (8),

where B may represent the amplitude of lactate formation rate in the muscle, k_3_ and k_4_ represent the time constants of appearance and disappearance of lactate in the blood and C depicts the limit value of blood lactate accumulation during maximal exercise.

t_alac_ reflects the time delay of the onset of lactate accumulation in the blood [16], so that ΔBLC (t_alac_)=0. Maximal rate of lactate accumulation (v̇La_max_ mmol l^−1^ s^−1^) was calculated by:

$$\dot{\text{V}}{\text{La}}_{\text{max}}:=\underset{\text{t}\in ]{\text{t}}_{\text{alac}};60}{\text{max}}\dot{\text{v}}\text{La}\left(\text{t}\right)$$ (9),

where the function of the lactate accumulation rate was the derivative of ΔBLC(t).

The kinetics of oxygen uptake during the 60 s-sprint were described by Barstow & Molé [37]:

$$\dot{\text{V}}{\text{O}}_{2}\left(\text{t}\right)=\dot{\text{V}}{\text{O}}_{2\text{A}}\cdot \left(1{\text{-e}}^{\frac{\text{-t}}{{}^{\text{τ}}\text{VO2}}}\right)+\dot{\text{V}}{\text{O}}_{2}\left(0\right)$$ (10),

where V̇O_2_(t) represents the oxygen uptake at time t. V̇O_2_(0) is the initial oxygen uptake or starting value immediately before the start of exercise. V̇O_2A_ defines the amplitude or denotes the change between the initial and current oxygen demand. To investigate the dependence of oxygen saturation on oxygen availability, the time-dependent quotient of changes in oxygen saturations (ΔSmO_2_(t) = SmO_2_(t)/SmO_2_(t + 1)100 [%]) and changes in oxygen uptake (ΔV̇O_2_(t) = V̇O_2_(t)/V̇O_2_(t + 1))100 [%]) was calculated.

### Statistical analyses

All data were checked for normality using the Shapiro-Wilk test and are presented as mean ± SD. The Pearson product-moment correlation test was used for analysing interrelationships between variables. Pearson’s correlation coefficient r (small; r ≥ 0.1; medium: r ≥ 0.3; large: r ≥ 0.5) was employed as a measure of effect size. Statistical significance was set at an alpha level of < 0.05. Linear and non-linear regression analysis was used to study the relationship between data. The least squares method was used in the regression analysis to determine the model parameters and time delays of the various model functions. The absolute difference was used to determine the bias of measurements. The quality of the regression analyses was examined by calculating the coefficient of determination . Mathematical analysis and statistical tests were processed using IBM SPSS Statistics version 24 for Windows (IBM Corp., Amonk, NY, USA), Office Excel 2016 (Microsoft Corporation, Redmond, WA, USA) and MATLAB 9.10.0 R2021a (The MathWorks, Inc., Natick, MA, USA).

## RESULTS

**FIG. 1:**
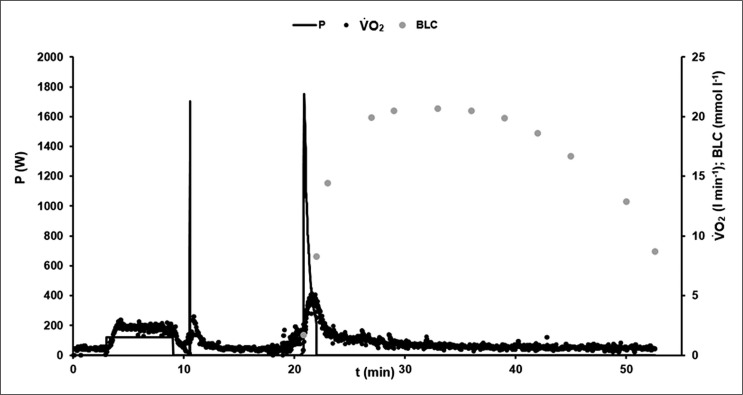
Example of the time-course of power output P (W), oxygen consumption V̇O_2_ (l · min^−1^), and blood lactate concentration BLC (mmol · l^−1^) during the 60-s cycling sprint test protocol.

Figure 1 illustrates the time-course of power output, oxygen uptake and blood lactate concentration in an exemplary 60-s test.

After the initial acceleration phase, the athletes reached the specified cadence of 120 rpm within the first 4 s in all sprint tests. To control the quality of the performance within the tests, individual fatigue-free F/v profiles were compared. No statistically significant differences were found.

Considering only the best F/v profile of each athlete (the profile with the highest calculated maximal power output), mean maximal force was 1343 ± 107 N and mean maximal cadence was 300 ± 15 rpm. The slope of the F/v profile was a = -4.51 ± 0.53 N rpm^−1^. The coefficient of determination R^2^ for the F/v profile was calculated to be ≥ 0.99 for all athletes. The time-point immediately before the first systematic deviation from the fatigue free F/v profile was located at t_Ff_ = 2.06 ± 0.32 s.

Mean power output, ΔBLC and the model parameters of EPOC(t) of the different sprint tests are shown in Table 1.

**TABLE 1:** Mean power output (P_mean_), mean blood lactate accumulation (ΔBLC), mean parameters of excess post exercise oxygen consumption EPOC(t) and mean phosphagen energy supply (W_PCr_) of the 3-, 8-, 12- and 60-s sprint test (n = 9).

Parameters	3 s	8 s	12 s	60 s
	M ± SD	M ± SD	M ± SD	M ± SD
**P_mean_(W)**	1151 ± 116	1395 ± 109	1371 ± 102	715 ± 42
**ΔBLC (mmol · l^-1^)**	0.66 ± 0.25	4.73 ± 0.65	8.16 ± 0.90	17.70 ± 2.62
**A_FC_ (ml · min^-1^)**	1985 ± 436	2740 ± 381	2878 ± 501	3032 ± 447
**τ_a_ (min)**	0.82 ± 0.14	0.84 ± 0.12	0.84 ± 13	0.85 ± 13
**B_SC_ (ml · min^-1^)**	195 ± 167	418 ± 133	577 ± 119	981 ± 298
**τ_b_ (min)**	5.72 ± 2.52	6.46 ± 3.38	8.03 ± 3.10	9.36 ± 5.11
V̇**O_2Base_(ml · min^-1^)**	391 ± 58	391 ± 43	411 ± 58	532 ± 106
**W_PCr_ (kJ)**	31.83 ± 6.98	46.50 ± 3.79	51.24 ± 9.52	53.73 ± 9.96

Abbrev.: P_mean_: Mean power output; ΔBLC: Blood lactate accumulation; A_FC_: Amplitude of the fast component of EPOC(t); τ_a_: Time constant of the fast component of EPOC(t); B_SC_: Amplitude of the slow component of EPOC(t); τ_b_: Time constant of the slow component of EPOC(t); V̇O_2Base_: Oxygen consumption at rest as the limit value of EPOC(t); W_PCr_: Anaerobic alactic energy supply calculated based on the fast component of EPOC(t).

**FIG. 2:**
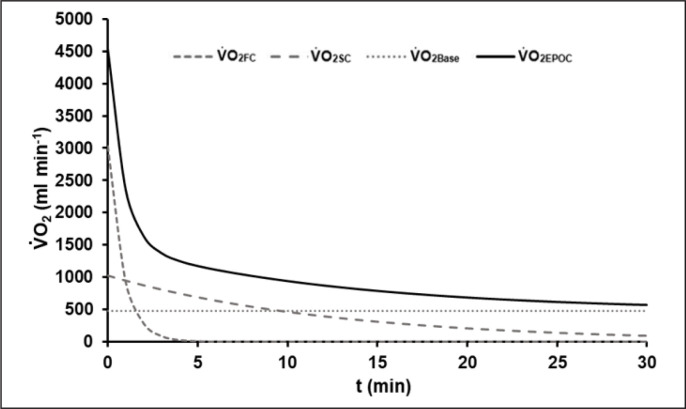
Mean model function of EPOC(t) after the 60-s sprint test with its fast (V̇O_2FC_) and slow components (V̇O_2SC_) and the asymptotic resting level of oxygen uptake (V̇O_2Base_). The fast component corresponds to the amount of energy gained from the dephosphorylation of high-energy phosphates during exercise.

The average R^2^ of all EPOC-models was 0.90 ± 0.03. The mean EPOC(t) of a 60-s test with the different components is illustrated in Figure 2.

**FIG. 3:**
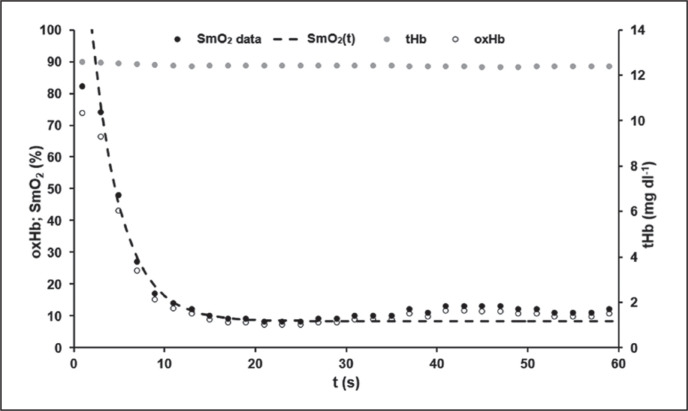
A typical example of the data for the total mass of haemoglobin and myoglobin (tHb) with its oxygenated portion (oxHb) and for the muscle oxygen saturation data (SmO_2_) with the calculated model SmO_2_(t) during the 60-s sprint test. SmO_2_(t) showed a very good description of the SmO_2_ raw data until the middle of the test. After that, a slight increase in SmO_2_ could be observed despite the athlete’s maximal effort. The level of tHb was almost constant throughout the sprint. This phenomenon could be observed in all athletes.

Mean model parameters of oxygen desaturation (equation 3) of the VL were SmO_2Base_ = 75.13 ± 8.60%, SmO_2A_ = 65.94 ± 9.36% and τ_SmO2_ = 2.93 ± 0.65 s. An R^2^ > 0.99 was calculated. The time-point at which modelled ΔSmO_2_(t) started to increase systematically above 100% and therefore SmO_2_ values differ from the model function was t_resat_ = 32.60 ± 4.96 s. An example of SmO_2_ data and the determined model SmO_2_(t) of an athlete’s 60-s sprint test is shown in Figure 3.

tHb values ranged between 12.95 ± 0.33 mg dl^−1^ and 12.74 ± 0.31 mg dl^−1^ with 0.22 mg dl^−1^ as the largest difference observed. The maximal value of tHb was reached at 24.8 ± 22.8 s, the minimal value at 15.3 ± 16.0 s, not indicating any time-dependent systematics.

Mean parameters of oxygen uptake kinetics were V̇O_2A_ = 3.70 ± 0.44 l min^−1^, τ_V̇O2_ = 14.73 ± 3.00 s and V̇O_2_(0) = 0.68 ± 0.20 l min^−1^ with a mean R^2^ of 0.85 ± 0.10.

The time-dependent quotient of changes in oxygen saturation and changes in oxygen uptake (ΔSmO_2_(t) ΔV̇O_2_(t)^−1^) was >> 1 in the relevant period of exercise (t_resat_ < t ≤ 60 s).

Based on W_PCr_ of the different sprint tests, the parameters of equation 6 to describe the time-dependent energy supply of the phosphagen energy system W_PCr_(t) were W_PCrTOT_ = 53.02 ± 9.30 kJ and τ_PCr_ = 3.23 ± 0.67 s with an R^2^ of > 0.98.

**FIG. 4:**
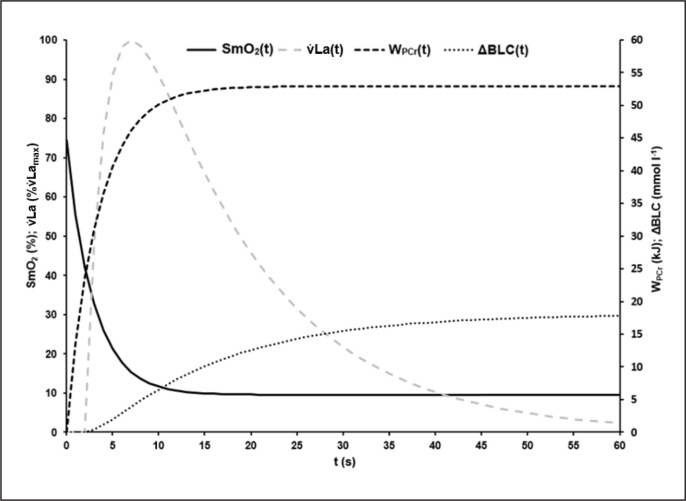
Mean time-dependent O_2_ saturation of the vastus lateralis muscle (SmO_2_(t)), mean time-dependent blood lactate accumulation (ΔBLC(t)) and mean time-dependent phosphagen energy supply (W_PCr_(t)) during the 60-s sprint test. By visual comparison, a high correlation of the kinetics can be assumed, which could be confirmed statistically.

Based on ΔBLC of the different sprint tests, the parameters of equation 8 describing the time-dependent blood lactate accumulation ΔBLC(t) were B = 1.59 ± 0.40 mmol · l^−1^ · s^−1^, k_3_ = 0.07 ± 0.02 s, k_4_ = 0.62 ± 0.28 s and C = 18.78 ± 2.99 mmol · l^−1^ with an R^2^ of > 0.99. t_alac_ was determined at 2.09 ± 0.41 s. Mean maximal rate of lactate accumulation was v̇La_max_ = 0.95 ± 0.18 mmol · l^−1^ · s-^1^.The mean kinetics of SmO_2_(t), W_PCr_(t), ΔBLC(t) and La(t) in the 60-s sprint tests are shown in Figure 4. SmO_2A_ showed a significant strong correlation with SmO_2Base_ (r = 0.911; p < 0.002). τ_SmO2_ was positively correlated with τ_PCr_ (r = 0.790, p < 0.012), v̇La_max_ (r = 0.768, p < 0.017) and t_alac_ (r = 0.822, p < 0.001). t_Ff_ was positively correlated with τ_SmO2_ (r = 0.885, p < 0.001) and τ_PCr_ (r = 0.781, p < 0.008). Furthermore, a negative correlation of τ_SmO2_ and PR_max_ (r = -0.670, p < 0.049) was found.

**FIG. 5:**
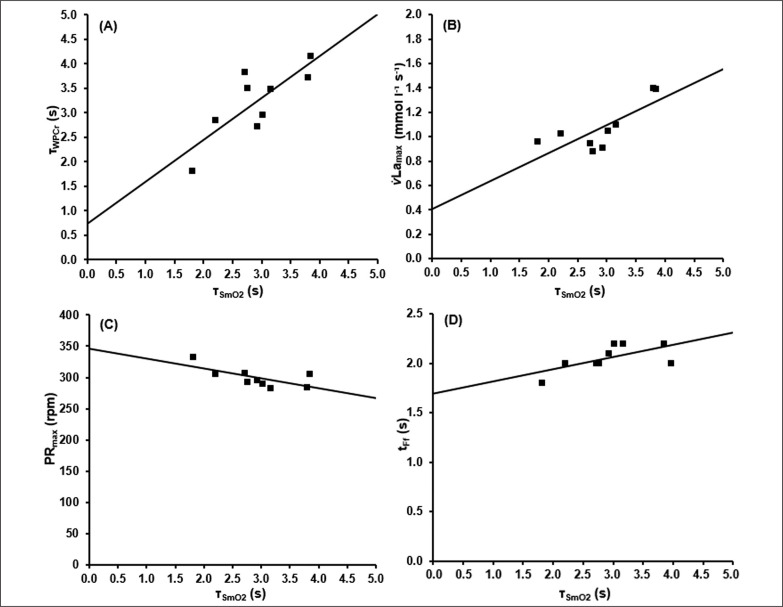
Results of the correlation analysis between the time constant of SmO_2_(t)(τ_SmO2_) and A) the time constant of W_PCr_(t) (τ_PCr_), B) the maximal rate of blood lactate accumulation (v̇La_max_), C) the maximal pedalling rate (PR_max_) and D) the end of the fatigue-free state (t_Ff_).

The associations between these parameters are shown in Figure 5.

**FIG. 6:**
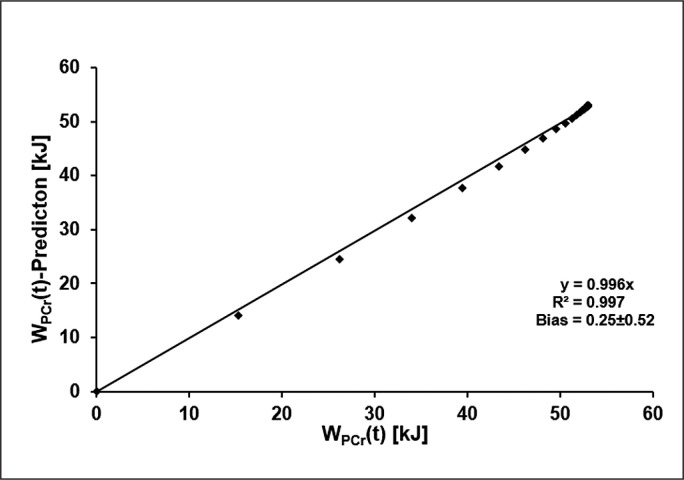
Deviation of the model of the time-dependent amount of alactic energy predicted using the mean time constant of SmO_2_(t) from the mean original model of W_PCr_(t) in each second during the 60-s sprint with bias and standard deviation (SD).

Using the mean time constant of SmO_2_(t) in combination with W_PCrTOT_ to estimate W_PCr_(t) results in a maximum error of 1.91 kJ. The results of the linear regression analysis comparing the two functions at each second during the 60-second sprint are shown in Figure 6.

## DISCUSSION

In accordance with Hamaoka et al. [2, 11], Jones et al. [38], Hamaoka & McCully [39] and Vasquez-Bonilla et al. [40], our results demonstrate a significant correlation between desaturation behaviour of SmO_2_ and the time-dependent change in PCr. In addition, significant correlations of SmO_2_(t) with maximal pedalling rate and maximal lactate accumulation rate were found.

All models show excellent quality, qualifying them as suitable for describing the time-dependent behaviour of SmO_2_ desaturation (SmO_2_(t)), phosphagen energy supply (W_PCr_(t)), blood lactate accumulation (ΔBLC(t)) and oxygen uptake (V̇O_2_(t)) in an isokinetic 60-s all-out test on a cycle ergometer.

W_PCr_(t) was described by a mono-exponential function that shows a steep increase in phosphagen energy contribution in the first seconds of maximal exercise. After approximately 10 s, the rate of phosphagen energy contribution is significantly reduced and at approximately 12 s W_PCr_(t) reaches a quasi-steady state close to the maximum, which is mathematically reflected by the limit value of the function. According to the results of di Prampero and colleagues [41] the calculated amount of total W_PCr_ of ~ 53 kJ, assuming an active muscle mass of circa 30% in cycling exercises [42], corresponds to about 26 mmol phosphagen split per kg active wet muscle. This value is in the same order of magnitude as the total phosphagen content of the resting muscle [16]. The time to maximum is in line with previous observations. Barclay [13] reported that during maximal exercise, the energy received from the phosphagen system peaks after 10–15 s. Therefore, a glycolytic and mitochondrial contribution sufficient to maintain a quasi-steady state of re- and dephosphorylation of PCr in the working muscles is assumed at the time-point of near zero energy contribution of the phosphagen system. This state is also reflected in the desaturation behaviour of SmO_2_ up to ~30 s.

Similar to W_PCr_(t), SmO_2_(t) can be described mono-exponentially and reflects the PCr dephosphorylation behaviour well. SmO_2_(t) also shows a steep desaturation in the first few seconds of the test. After a subsequent decrease in the desaturation rate after approximately 10 s, SmO_2_(t) also approaches a plateau after approximately 12 s. Unlike W_PCr_(t), SmO_2_(t) eventually increases slightly after the initial steep desaturation from the beginning of the test (see Figure 3). Maximal voluntary effort was ensured retrospectively by applying our previously published method [29]. Even though individuals continued their efforts at maximal voluntary intensity, relative SmO_2_ extraction decreases.

The correlation of the time constants τ_SmO2_ and τ_PCr_ and the high agreement of the time courses indicate a close relationship between PCr dephosphorylation and SmO_2_ desaturation in maximal cycling sprints. This statement is supported in contemporary exercise physiological literature [4, 42, 43]. If the O_2_ stored in the tissue primarily serves PCr resynthesis between twitch contractions during exercise, the SmO_2_ desaturation rate should depend on the PCr dephosphorylation rate [7, 9]. This is in line with di Prampero [41] and Francescato and colleagues [44], who described a transfer of O_2_ from Hb on Mb on the working muscle with the onset of exercise. The differences of the time constants calculated in this study could be explained by measurement inaccuracies (especially in determination of the fast component of EPOC, which represents the greatest uncertainty in this study), the difference between local and systemic measurement or by the additional inhibiting influence of high glycolytic activity on PCr dephosphorylation state, whereas the shift of the cell pH to acidic due to high ATP hydrolysis additionally favours O_2_ dissociation [19].

Our results indicate that the time-point immediatley before the first systematic deviation from the fatigue-free F/v profile could reflect the half-life SmO_2_ and W_PCr_ and could indicate the onset of accumulation of lactate in the blood. Assuming an activation of glycolytic and oxidative ATP resynthesis after a depletion of 10–12 mmol PCr [16], which, applied to our data, corresponds to approximately 40–45%, the temporal proximity of these parameters seems plausible.

ΔBLC(t) was described by a bi-exponential function using t_alac_ to describe the delayed onset of blood lactate accumulation [41]. After a mean t_alac_ of 2.09 s, ΔBLC(t) rises steeply in an approximately linear manner until the tenth to twelfth second of exercise. After 10–12 s the increase in blood lactate accumulation is reduced, reflecting a sudden decrease of glycolytic activity caused by pH reduction in the active muscle cells [12, 20]. Extrapolated ΔBLC for 30 s of maximum effort is in accordance to the values reported by Beneke et al. [45] and Leithäuser et al. [46], which provides confirmation of model validity.

**FIG. 7:**
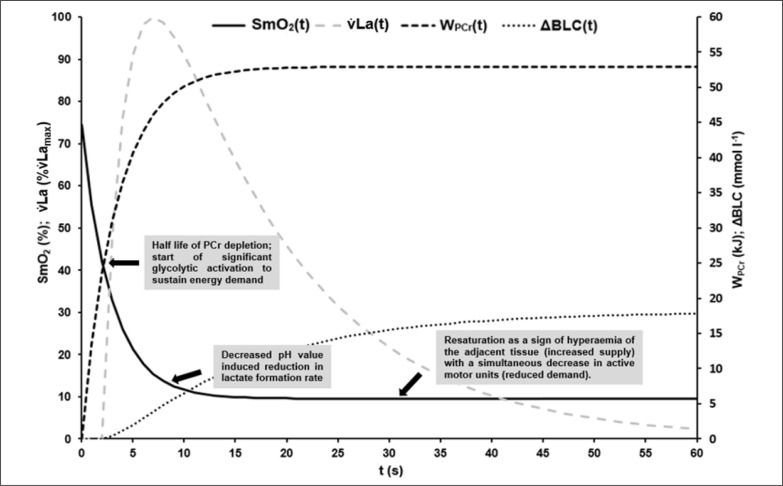
Visualisation of the mean time courses of oxygen saturation SmO_2_(t) [%], time-dependent anaerobic alactic energy contribution W_PCr_(t) [kJ], time-dependent blood lactate accumulation ΔBLC(t) [mmol · l^−1^] and resulting lactate formation rate v̇La(t) [mmol · l^−1^ · s^−1^] with suggested interpretation of characteristic point in times: At the onset of maximal exercise the rate of PCr split reflects the energy demand of the working muscle [16, 17]. About the have of the PCr stored in the muscle can be depleted without significant changes in ATP depletion rate and ATP concentration in the working muscle [16]. Assuming that glycolytic and oxidative ATP resynthesis is activated by the cell’s ADP concentration or phosphorylation deficit, a subsequent rapid increase in ADP or Cr will then activate glycolytic and mitochondrial ATP resynthesis to compensate for an increased rate of dephosphorylation [12]. The higher the rate of glycolytic and mitochondrial rephosphorylation, the slower the rate of PCr dephosphorylation subsequently. Due to the comparatively low glycolytic and the even lower oxidative ATP resynthesis rate – especially at the beginning of maximal exercise – the energy flow in the muscle cell continuously decreases, which leads to a corresponding continuous decrease in muscle performance. Although oxidative phosphorylation subsequently continues to increase until the cardiopulmonary system has adapted to the maximal energy demand, an exponential decrease in maximal power output can be observed [29]. This is due to a successive decrease in glycolytic energy flux with decreasing cell pH, resulting in a decrease in active muscle mass when energy flux falls below the minimum demand required for excitability and muscle contraction, especially in dominant phosphagenic and glycolytic type IIx muscle fibres. If SmO_2_ always reflects the balance between oxygen demand and supply of a muscle, the sudden resaturation of SmO_2_ at the midpoint of the sprints could indicate a decrease in total PCr restoring demand and therefore a decrease in the active muscle mass (see Figure 3).

The onset of sudden significant reduction of lactate accumulation seems to coincide with the time-point when PCr dephosphorylation and SmO_2_ desaturation approach their respective plateaus (see Figure 7). A positive correlation between the maximal lactate accumulation rate and the time constant of O_2_ desaturation of the VL was observed. The higher v̇La_max_, the higher τ_SmO2_ and the longer it takes for the SmO_2_ desaturation to reach a steady state. Considering the correlation of SmO_2_ desaturation and PCr dephosphorylation, a higher v̇La_max_ representing a higher rate of PCr replenishment via glycolysis between twitch contractions can be assumed, reducing the need for oxidative rephosphorylation of PCr due to a smaller initial alactic debt in the dominant working muscle.

Hautier et al. [26] and Hansen et al. [47] found a positive correlation in the proportion of fast glycolytic IIx muscle fibres in the VL with maximal pedalling rate. A significant correlation between the maximal pedalling rate and the time constant of SmO_2_ desaturation was found. As fast contracting glycolytic muscle fibres have the highest ATPase activity, highest maximal energy flow rate and the fastest dephosphorylation of PCr [48, 49], the correlation between τ_SmO2_ and τ_PCr_ as well as PR_max_ and the muscle fibre spectrum seems plausible.

Although the potential for a high rate of glycolysis increases with the proportion of fast fibres [25], no correlation between PR_max_ and v̇La_max_ was found within this sample of elite power athletes. This result suggests that the specific enzyme activity required for high glycolytic energy flow is not exclusively morphologically determined, but represents a specific training adaptation.

The previously described sudden resaturation of SmO_2_ in the VL after approximately 30 s of maximal exercise could be a multifactorial phenomenon reflecting the onset of a decrease in the active muscle fibre spectrum in the muscle, the delayed response of the cardiovascular system to rapid changes in peripheral energy demand and the haemodynamics of the capillary beds to maintain oxygen supply to the muscle.

Thomas and Victor [19] reported that a kind of contraction-induced sympathetic ischaemic state prevails in the muscles, which does not allow the influx of oxygenated blood for the very first seconds of maximal exercise. If this was the case, resaturation of SmO_2_ in the working muscle would not be possible initially. With accumulation of metabolites in the interstitial fluid (K+ , lactate) [40], vasomotor relaxation occurs, mediated by the endogenous vasodilator nitric oxide (NO), attenuating sympathetic vasoconstriction in active muscles and contributing to greater hyperaemia during exercise [19]. Although such an effect cannot be excluded, our results do not indicate any significant reduction of blood flow and thus no restriction of oxygen availability during exercise due to temporary ischaemic states. Thus, a non-significant and time-related non-systematic change in tHb by a maximum of < 2% could be detected.

Although V̇O_2_(t) increased exponentially from the initial value of ~15% to ~85% of the athletes’ V̇O_2max_ during the 60-s sprint, oxygen uptake kinetics cannot explain the sudden resaturation of SmO_2_ after ~ 30 s of exercise, which, at a nearly constant tHb, has to represent a resaturation of Hb and Mb in the VL.

The V̇O_2_(t) time constant (τ_V̇O2_ ~ 15 s) leads to a steep increase in oxygen uptake during the first 15 s of maximal sprinting. After 30 s of exercise, about 90% of the V̇O_2peak_ was reached. In the second half of the sprint, oxygen uptake only increased by < 10% with increments of < 0.75% s^−1^.

The relationship of resaturation rate and changes in oxygen uptake indicates a decoupling of resaturation and oxygen uptake in the second half of exercise (ΔSmO_2_(t) > 100%, ΔV̇O_2_ (t) < 100% 30 < t ≤ 60 s). From our results, we suggest that the resaturation during exercise is neither an effect of blood flow restriction nor a delayed response of the cardiovascular system to rapid changes in peripheral energy demand.

Considering the exponential decrease in maximal power output [29] and the reported exponential increase in blood lactate concentration, it can be speculated that the progressive increase in resaturation of Hb and Mb from mid-exercise onwards may reflect the onset of rephosphorylation of PCr in fatigued high-glycolytic IIx fibres, which no longer contribute to power output [29, 50].

The assumption that resaturation during maximal exercise is an indication of a decrease in the number of working motor units in the dominant working muscles is also supported by the findings of Vasquez-Bonilla et al. [40]. In a repeated sprint protocol, a decrease in desaturation amplitude was observed in elite female football players with a simultaneous decrease in sprint performance in each bout. This is consistent with our observations of NIRS signalling during repeated cycling sprints.

Finally, Figure 7 illustrates the possible interpretation of SmO_2_ kinetics based on the results of our study.

## LIMITATIONS

The study is based on a considerable amount of mathematical modelling of physiological responses during maximal exercise. Despite the high quality of the individual models and plausibility of the overall modelling, the simplifying character of a model in favour of the identification of interrelationships and systematic behaviour has to be considered.

The models are based on data points derived from several maximal sprint tests. The validity of the models requires a high reliability of performance, metabolic stress and their measurements. Despite our efforts to standardise the tests and ensure similar test conditions, it must be pointed out that it is an indirect reconstruction of the metabolic response during the 60-s effort, so that misinterpretation cannot be excluded completely.

Due to the limited number of participants, elite training status and the possible sources of error presented, further investigations are required to check our results, especially in different cohorts. When recruiting participants, care must be taken to ensure a sufficient level of capability and proficiency, as the method used here requires the athletes to sustain maximal neuromuscular performance. If this performance is below the time-dependent maximum, metabolism in the fibres of the active muscles may vary, altering total metabolic response and its systematics.

## PRACTICAL APPLICATIONS

For practical application, the SmO_2_ kinetics of the muscle with the highest desaturation within the main propulsive/working muscles can be used as a representation for the PCr dephosphorylation state of the system.

By combining the value of total alactic energy derived from oxygen uptake of the fast component of EPOC with the time constant of SmO_2_(t) derived from NIRS measurements, PCr dephosphorylation kinetics can be estimated on the basis of a single sprint with an error < 2 kJ.

A direct measurement of the lactate accumulation rate based on NIRS measurements does not seem to be possible, but the time interval from the onset to the maximum activation of the lactate accumulation rate can be represented by NIRS measurements.

## CONCLUSIONS

Our results indicate that SmO_2_ kinetics of the dominant working muscle reflect the time-dependent phosphagen energy contribution and the dephosphorylation of high-energy phosphates in a 60-s maximal sprint in elite track cyclists. The rate of SmO_2_ desaturation is dependent on the maximal pedalling rate and the maximal lactate accumulation rate reflecting the maximal glycolytic energy flux. While higher PR_max_ accelerates desaturation, higher v̇La_max_ reduces it. The half-life of SmO_2_ represents the end of the fatigue-free state and could indicate the beginning of accumulation of lactate in the blood. Reaching an SmO_2_ plateau after maximal desaturation seems to be temporally associated with a reduction in lactate accumulation rate.
